# Methylcobalamin Deficiency Presenting as Thalamic Syndrome in the Elderly: Association or Chance?

**DOI:** 10.7759/cureus.52761

**Published:** 2024-01-22

**Authors:** Varun Daiya, Abhinav Ahuja, Sunil Kumar, Sourya Acharya

**Affiliations:** 1 Internal Medicine, Jawaharlal Nehru Medical College, Datta Meghe Institute of Medical Sciences (Deemed to be University), Wardha, IND; 2 Internal Medicine, Geriatric Medicine, Critical Care, and Palliative Care, Jawaharlal Nehru Medical College, Datta Meghe Institute of Medical Sciences (Deemed to be University), Wardha, IND; 3 Internal Medicine and Endocrinology, Jawaharlal Nehru Medical College, Datta Meghe Institute of Medical Sciences (Deemed to be University), Wardha, IND

**Keywords:** macrocytic anemia, paresthesia, psychosis, neuropsychiatric functioning, homocysteine levels, geriatric population, vitamin b12 deficiency

## Abstract

Vitamin deficiency is common in the geriatric population and is responsible for majorly imbalanced hematological, neurological, and neuropsychiatric functioning. Methylcobalamin deficiency or vitamin B_12_ deficiency can be underestimated in some cases and be misdiagnosed as other illnesses, such as thalamic syndrome. Timely diagnosis of this deficiency is essential, especially in the geriatric population, as it might cause irreversible structural brain damage. This is also presented as elevated levels of homocysteine and methylmalonic acid. Clinically, it presents with the following symptoms: lower sensitivity levels to touch and light, psychosis, paresthesia, anemia, imbalance, fatigue, cognitive disturbances, difficulty remembering, and confusion. Symptoms are usually progressive and worsen over a period of time. In this case report, we present the case of a 62-year-old male with clinical symptoms of numbness and tingling in the right side of the body. The neurological presentations resembled left thalamic infarct, but the underlying reason was methylcobalamin deficiency.

## Introduction

The human nervous system is highly dependent on dietary nutrients available from food, which is also responsible for normal functioning. Vitamins play a vital role in the development of the human nervous system. Vitamin deficiencies are quite commonly observed in many individuals today due to unhealthy food choices and an imbalanced lifestyle [1]. This is commonly observed in the geriatric population as they are dependent on others for their nutritional needs. Methylcobalamin or vitamin B_12_ deficiency is one of the most commonly observed conditions that interferes with normal physiological functioning. Vitamin B_12_ is a group of corrinoids that are essential for the normal functioning of many enzymes that carry out numerous essential biological activities in humans [2]. It is found to be deficient in many individuals in present times, attributed to unhealthy dietary habits. This deficiency is commonly observed in individuals of old age due to food malabsorption. Clinical symptoms presented due to its deficiency can be majorly hematological and neurological [1-3]. Paresthesia is commonly observed along with peripheral nerve damage, which is reported to be painful and co-occurring with ataxia in varying degrees. Methylcobalamin deficiency is reported to be presented with an initial involvement of the hands, later extending to the limbs and other parts of the body [3,4]. Vitamin B_12_ levels below 200 pg/mL in individuals are considered deficient. It is reported in approximately 2.5-26% of individuals. It is reported to affect all age groups equally, but the elderly population is more susceptible [1]. It has also been observed in vegans and a few individuals due to the deficiency of intrinsic factors which can be due to ageing. Due to ageing and the deficiency of intrinsic factors, methylcobalamin is crucial in homocysteine metabolism as methionine synthase, an enzyme found in the cytoplasm, induces the transfer of a methyl group from methyltetrahydrofolate from homocysteine to methionine and thus aids in the regeneration of methionine for protein synthesis and helps to avoid the accumulation of homocysteine. Vitamin B_12_ deficiency also correlates with increased methylmalonic acid and homocysteine levels [3]. The classical presentation of this deficiency in older adults includes psychotic, neurologic, cognitive, and macrocytic (anemia). It has also been found to be associated with irreversible brain damage or structural changes in the brain, especially in the geriatric population. High homocysteine levels play an important role in the pathophysiology of stroke. Endothelial damage develops depending on the status of high homocysteine levels, and this triggers other thromboembolic events. B group vitamins, especially folic acid and vitamin B_12_, are co-factors for homocysteine metabolism. The lack of these vitamins in the body leads to the degradation of homocysteine metabolism and elevated levels of homocysteine. Thus, high homocysteine levels and endothelial injury can be reduced by including folic acid and vitamin B_12_ in the diet, thereby reducing the risk of stroke. In case of severe deficiency, it has also been linked to spinal cord degeneration [4]. 

Common dietary sources of vitamin B_12_ are dairy products, eggs, and meat [1,4]. Vitamin B_12_ deficiency is known to cause subacute combined degeneration of the spinal cord in which dorsal column involvement is seen, which leads to impaired tactile discrimination, proprioception, and vibration sense. Lateral corticospinal dysfunction causes muscle weakness, hyperreflexia, and spasticity spinocerebellar tract degeneration, causing gait abnormalities in the form of sensory ataxia. Visual difficulty, mental problems, pancytopenia, sensory abnormalities, and general weakness clinically manifest it. Clinical scores of severity of subacute combined degeneration and vitamin B_12_ deficiency were found to be correlated [5]. However, timely diagnosis of this deficiency is essential for the prevention of irreversible neurological damage, especially in the geriatric population. The thalamus is vital in sensory functions and the normal functioning of motor and cognitive systems which maintains light, temperature, pain, and touch sensitivity in our body along with the management of the informational flow in motor, visual, and auditory channels [6]. Clinical diagnosis is confirmed by physicians' evaluation of neuropsychological and neurological aspects in cases of thalamic strokes [7]. In this case report, we have highlighted thalamic syndrome in an elderly patient who presented with thalamic syndrome due to methylcobalamin deficiency, which has not been reported earlier.

## Case presentation

A 62-year-old male presented with complaints of tingling and numbness in the right upper limb and lower limb for one month. The patient also complained of a burning type of pain in both upper and lower limbs for 10 days which aggravated on bathing with cold water. His symptoms started with tingling and numbness in the right side of the face and upper limb, which later expanded to most of the right side of the body. The patient was evaluated; however, vitamin B_12_ levels were not checked, and he was managed conservatively to which he did not respond. The patient was vegetarian by diet. There was no history of any neurological disorders such as confusion and seizures and other clinical symptoms such as vomiting, weakness, and fever. He did not have a history of any significant illnesses such as hypertension, diabetes, and ischemic heart disease. He was a non-smoker and non-alcoholic with a moderately built physique with a body mass index of 22. Physical examination revealed a pulse rate of 88 beats per minute and blood pressure of 140/90 mmHg. Central nervous system examination revealed an absent ankle reflex, impaired pinprick, and temperature sensations suggestive of peripheral neuropathy. Other neurological examinations were within normal limits. Laboratory parameters of the patient at presentation are presented in Table 1.

**Table 1:** Laboratory parameters of the patient INR: international normalized ratio

Parameter	Patient's values	Normal range
Hemoglobin	12.7 gm%	13-17 gm%
Total leukocyte count	7200 cells/cu mm	4,000-10,000 cells/cu mm
Total platelet count	1.75 lakh/cu mm	1.5-4.1 lakh/cu mm
Mean corpuscular volume	106 fL	83-101 fL
Homocysteine	15.24 mmol/L	6.6-14.8 mmol/L
Vitamin B_12_	184 pg/mL	300-900 pg/mL
Activated partial thromboplastin time	30.5 sec	29.5 sec
Prothrombin time	12.1 sec	<20 sec
INR	1.01	1-1.5
Urea	22 mg/dL	19-43 mg/dL
Creatinine	0.8 mg/dL	0.66-1.25 mg/dL
Serum sodium	135 mmol/L	135-145 mmol/L
Serum potassium	4.0 mmol/L	3.5-5.1 mmol/L
Alkaline phosphatase	79 U/L	38-126 U/L
Alanine aminotransferases	21 U/L	<50 U/L
Aspartate aminotransferase	19 U/L	17-59 U/L
Albumin	4.6 g/dL	3.5-5 g/dL
Total bilirubin	0.3 mg/dL	0.2-1.3 mg/dL
Bilirubin conjugated	0.1 mg/dL	0-0.3 mg/dL
Bilirubin unconjugated	0.2 mg/dL	0-1.1 mg/dL

**Figure 1:**
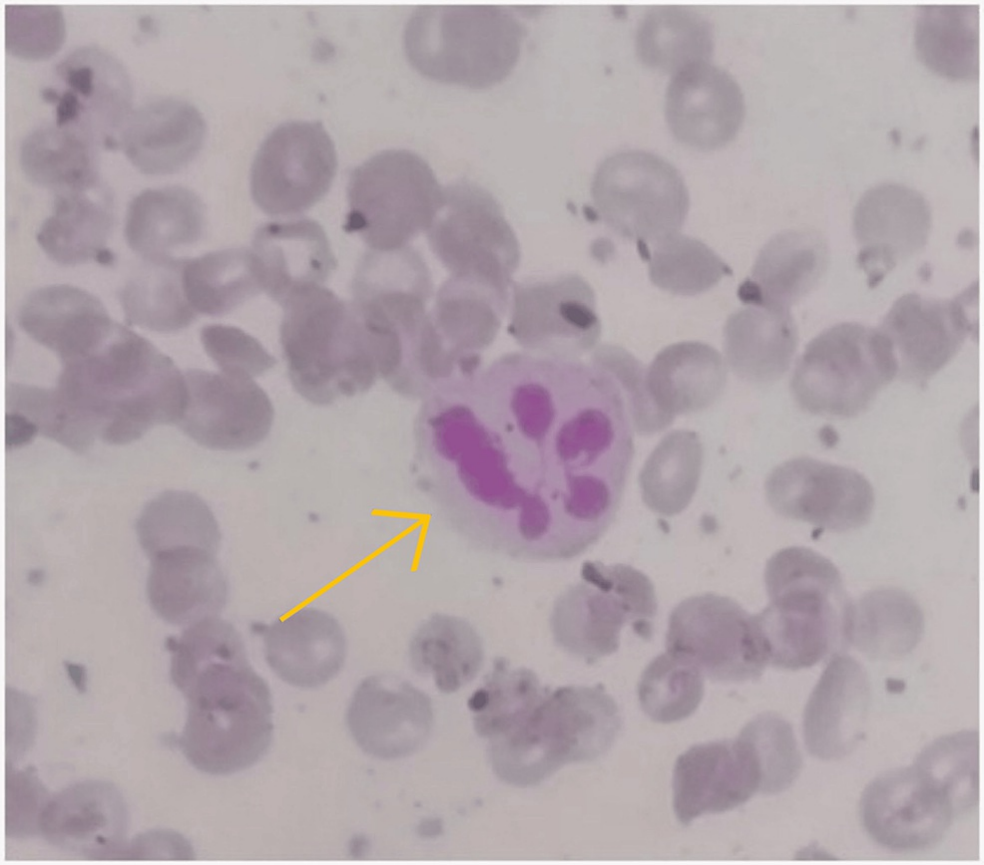
Histopathology slide representing hypersegmented neutrophil (yellow arrow)

Peripheral smear was suggestive of macrocytosis with hypersegmented neutrophils, as shown in Figure 1.

**Figure 2:**
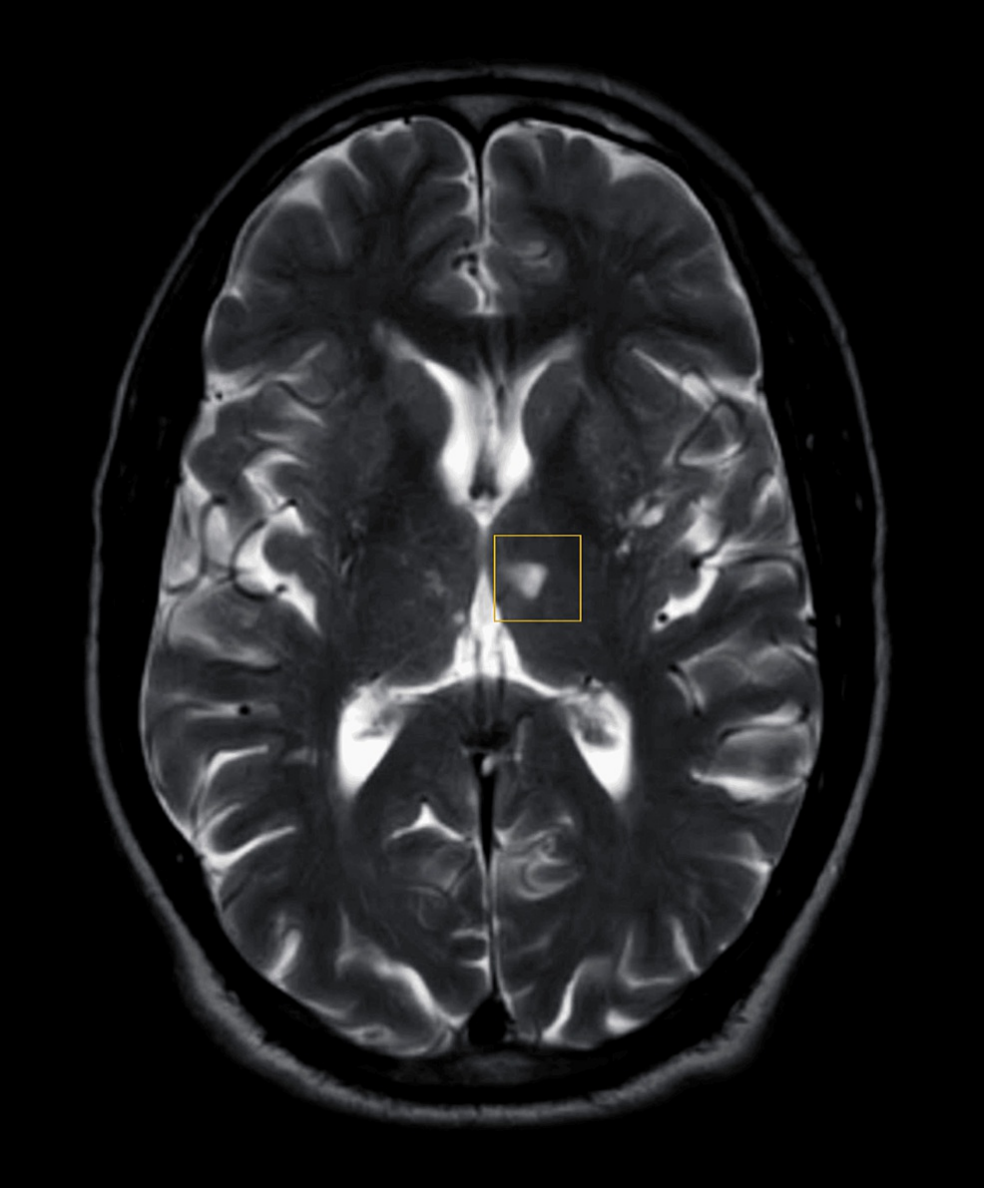
T2-weighted MRI scan showing left thalamic infarct The highlighted area of focus shows the thalamic infarct. MRI: magnetic resonance imaging

A nerve conduction study revealed neuropathy. Magnetic resonance imaging of the brain with magnetic resonance angiography and magnetic resonance venography was suggestive of acute evolving infarct in the left side of the body of the septum pellucidum and left thalamus, as shown in Figure 2.

Magnetic resonance angiography was suggestive of normal course and calibre in the bilateral internal carotid arteries, vertebral artery, circle of Willis, and its branches. No obvious filling defects and dilatation were noted. Magnetic resonance venography revealed normal internal jugular veins and sigmoid, transverse, and superior and inferior sagittal sinuses. 2D echo was normal with a left ventricular ejection fraction of 60% and good left ventricular systolic function. Bilateral carotid artery Doppler was found to be normal. Therapeutic intervention in the patient was carried out by IV administration of methylcobalamin 1000 mcg once daily for seven days and pregabalin 75 mg twice daily. The patient was previously taking amitriptyline for the pain but did not respond to it. He did respond to vitamin B_12_ supplementation, but it was not satisfactory so he was started on pregabalin for his symptoms. His symptoms started to resolve three weeks after the start of the treatment. The patient was discharged with an oral dose of methylcobalamin and pregabalin.

Follow-up and outcome

The patient has been under a regular outpatient department follow-up (thrice monthly) since his last visit, and to date, the chief complaints of the patient have been improved with no adverse event noted. Recent labs show normal-range mean corpuscular volume and normal-range vitamin B_12_ levels.

## Discussion

There is limited research available on acute thalamic syndrome and its clinical and neurological presentations. Unusual neurological symptoms that have been documented and may be associated with a shortage of methylcobalamin might include different motor and sensory impairments similar to syringomyelia and autonomic dysfunction [1,3]. Brain imaging may reveal severe white matter abnormalities in certain situations, which is consistent with demyelination and brain atrophy [8]. Methylcobalamin deficiency is also observed with elevated homocysteine levels, which poses an increased risk of thromboembolic events in cardiovascular and cerebrovascular systems. Thalamic infarct is rare [9,10]. In our case, we noted that unilateral stroke in the paramedian artery exhibited with posteromedian thalamic syndrome. This syndrome is clinically displayed as neuropsychological and neurological derangements. Thalamic syndrome is presented as a group of clinical presentations generally due to a dysfunctional or damaged thalamus. The thalamus is crucial in receiving and transmitting vital sensory information to the cerebral cortex. Common causes include stroke, vascular problems, tumours, infection, trauma, or degenerative diseases affecting the thalamus [2]. The syndrome manifests differently depending on the location and extent of thalamic damage [5,6]. Symptoms of thalamic syndrome might appear as sensory abnormalities, movement difficulties, dysesthesia, thalamic pain syndrome, and cognitive and emotional changes [11]. The diagnosis of thalamic syndrome involves a thorough neurological examination and imaging studies (such as magnetic resonance imaging or computed tomography scans) to determine the extent and cause of the thalamic damage [6,7]. The clinical findings in our patient corroborate the aforementioned findings with an unknown aetiology of the infarction. Our concern was focused on the tingling and numbness without any underlying cause of oculomotor or vestibular dysfunction. We discussed the hypothesis that the occurrence of infarction with methylcobalamin deficiency might be elevated homocysteine and methylmalonic acid levels in serum, resulting in varied neurologic disabilities.

## Conclusions

In conclusion, methylcobalamin deficiency can lead to severe irreversible damage, particularly to the nervous system, if not addressed promptly. This deficiency, attributed to its neurological and hematological symptoms, can at times be misdiagnosed as thalamic syndrome, which is also recognized by symptoms such as neuropsychological and neurological disturbances. It is also essential to recognize methylcobalamin deficiency on time due to its association with increased homocysteine levels, which can increase the risk of thrombosis and have significant adverse effects. Regular monitoring of vitamin B_12_ levels, proper nutrition, and early intervention are vital in preventing and managing vitamin B_12_ deficiency and its associated complications.
